# The mediating role of psychological resilience in the relationship between deep learning approach and mathematical creativity: integrating structural equation model and network analysis

**DOI:** 10.3389/fpsyg.2025.1697817

**Published:** 2025-11-27

**Authors:** Ziqi Zeng, Yayun Zhuo, Dieni Lin, Weiming He, Yingyi Liu, Wanlun Liu, Xiantong Yang

**Affiliations:** 1 School of Materials and New Energy, South China Normal University, Shanwei, China; 2 School of Foreign Studies, South China Normal University, Guangzhou, China; 3 Faculty of Psychology, Beijing Normal University, Beijing, China; 4 School of Psychology, Fujian Normal University, Fuzhou, China

**Keywords:** mathematical creativity, psychological resilience, learning approaches, deep learning, network analysis

## Abstract

**Background:**

Previous scholars have conducted a series of explorations on the relationship between learning approaches and mathematical creativity, but it remains unclear how deep learning approach predicts mathematical creativity. According to the componential theory of creativity, psychological resilience may be one of the mediating processes, but the network relationships among psychological resilience, learning approaches, and mathematical creativity are currently unclear.

**Methods:**

To clarify these relationships, we recruited 986 Chinese university students to complete questionnaire surveys, employing an integrated approach combining structural equation modeling and network analysis for the first time to reveal the mediating relationship and network relationships among learning approaches, psychological resilience, and mathematical creativity.

**Results:**

The study found that deep learning approach positively predicted mathematical creativity through the mediation of psychological resilience. In the network, node centrality demonstrates the strongest characteristics across three dimensions: strength, closeness, and expected influences, followed by psychological resilience, while deep learning methods exhibit the weakest characteristics. Therefore, compared to stimulating mathematical creativity at the motivational level, directly stimulating it from the volitional level like psychological resilience would be a more effective approach.

**Conclusion:**

The findings of this study contribute to helping educational practitioners understand the internal relationships among learning approaches, psychological resilience, and mathematical creativity.

## 1 Background

With the growing demand for innovative talent today, fostering students’ creativity has increasingly become an important goal in the field of education (Songkram and Chootongchai, 2020). Mathematical creativity is a crucial creativity factor in STEM education for cultivating contemporary university students’ innovative abilities (Newton et al., 2022) and is considered one of the core competencies for future employment and economic development (Tubb et al., 2020).

Existing research shows that mathematical creativity is predicted by multiple factors including cognitive, emotional, and personality traits. In cognitive aspects, both intelligence and general creativity have been proven to have positive correlations with mathematical creativity (Meier et al., 2021). In terms of emotions and traits, mathematical creative self-efficacy has an important impact on the development of mathematical creativity (Bicer et al., 2020), and mathematical imagination is closely connected with creative thinking in mathematics, all of which can stimulate mathematical creativity (Irakleous et al., 2022). However, learning approaches—an important motivational factor influencing creativity—lack research on their mechanisms of action on mathematical creativity. Nevertheless, exploring only motivational factors in the process of investigating creativity predict mechanisms is far from sufficient. Volitional factors play an indispensable supporting role, such as psychological resilience, which is a common psychological trait in creativity research (Li and Li, 2025). Although existing research has shown that resilience and creativity positively predict mathematical problem-solving ability (Suhendri, 2018), no study has explored the potential relationship between psychological resilience and mathematical creativity. Furthermore, many studies have shown that mathematical deep learning can significantly enhance students’ academic resilience, but fewer studies have explored its relationship with psychological resilience (Weißenfels et al., 2023). Academic resilience is essentially a domain-specific manifestation of psychological resilience (Seçer and Ulaş, 2020), while psychological resilience emphasizes an individual’s overall adaptability across a wide range of life situations and is therefore more universal (Stoffel and Cain, 2018).

Based on this, the present study focuses on adopting a novel perspective: taking learning approaches as an antecedent of motivation, and psychological resilience as a volitional mediator, exploring the relationships among them and mathematical creativity from this dual perspective, hoping to find better ways to stimulate mathematical creativity and facilitate researchers’ understanding of their deep internal connections.

## 2 Literature review

### 2.1 Mathematical creativity

Mathematical creativity is typically defined as the process of generating novel and useful solutions or insights in mathematical contexts (Bruhn and Lüken, 2023), primarily measuring fluency (the ability to rapidly generate a large number of relevant thoughts, solutions, or responses in specific mathematical contexts) (Tabach and Levenson, 2018), flexibility (the ability to handle mathematical problems from different angles, strategies, or perspectives, and to flexibly adjust problem-solving methods) (Munaji et al., 2025), and originality (the ability to propose unique and novel solutions to the same mathematical problem and to break free from conventional thinking limitations) (Ulusoy et al., 2025) across three dimensions, representing an extension of creativity into the mathematical domain.

### 2.2 Deep learning approach

Learning goal orientation has a significant positive relationship with creativity (Huang and Luthans, 2015). Specifically in mathematics, research has proposed that mathematical creative thinking ability and creative disposition should be considered core components of mathematical learning orientation (Khalil and Prahmana, 2023). It should be noted that both learning approaches and learning orientation are related to motivational factors. Learning orientation emphasizes intrinsic learning motivation choices (Sablić et al., 2021), but according to self-determination theory, motivation in the learning process is largely reflected in the choice of learning approaches (Kyndt et al., 2011). General learning approaches can be divided into deep learning and surface learning, where deep learning emphasizes deep understanding, critical evaluation, and application of knowledge, while surface learning focuses on memorizing facts and passive reproduction of content (Russell, 2004; Frăsineanu, 2013). Research shows that deep learners tend to actively explore, question, and integrate information, enabling them to transform knowledge innovatively and apply it creatively (Li et al., 2023). Additionally, deep learning approach is positively correlated with higher self-efficacy, while surface learning shows the opposite (Agarwal et al., 2000).

### 2.3 Psychological resilience

Psychological resilience is a key concept in psychology, referring to an individual’s ability to adapt proactively and maintain or quickly recover psychological and behavioral functioning in the face of adversity, trauma, or significant stressors (Shi et al., 2019). In research with Chinese participants, psychological resilience can be divided into three key dimensions: tenacity, strength, and optimism (Yu and Zhang, 2007). Tenacity refers to an individual’s ability to remain calm, persevere, and refuse to give up in the face of difficulties and challenges, emphasizing sustained resistance to adversity (Liang and Wang, 2008). Strength reflects an individual’s subjective ability to recover from setbacks and become stronger, focusing on self-improvement through coping with stress (Han et al., 2023). Optimism reflects an individual’s focus on the positive side of things and manifests as a positive anticipation for coping with adversity (Sun et al., 2023). In summary, tenacity, strength, and optimism together constitute the core elements of psychological resilience. They are interrelated and together determine an individual’s ability to adapt and thrive in the face of adversity. This dimensional categorization has been widely used in research with Chinese participants (Zou et al., 2024).

### 2.4 The relation between deep learning approach and mathematical creativity

Jean Piaget believed that learning is a constructive process where individuals combine new knowledge with their existing cognitive schemas through assimilation and accommodation, generating new schemas and achieving new equilibrium (Abella et al., 2020). Deep learning is an understanding-based learning approach that requires learners to actively integrate new and old knowledge. This cognitive flexibility is one of the core elements of creativity (Vedenpää and Lonka, 2014) and aligns with Piaget’s theory of cognitive development stages. When students use deep learning approach to explore the essential logic of mathematics, they can more flexibly reconstruct problems and may demonstrate higher thinking fluency and originality, thereby enhancing mathematical creativity (Bicer et al., 2024). Interestingly, students with high mathematical creativity also typically tend to question conventional solutions and actively construct new knowledge networks to achieve deeper understanding (Singer, 2018; Kozlowski and Si, 2019). Conversely, relying on repetitive practice and rote memorization leads to fragmented student knowledge, greatly inhibiting their associative and knowledge reconstruction abilities (Supandi et al., 2021), making them more prone to rigid thinking in open mathematical problems (Tampa and Ruslan, 2021). Deep learning approach has close logical connections with mathematical creativity and may be one of the key factors in stimulating mathematical creativity. Based on this, the present study hypothesizes H1: Deep learning approach positively predicts mathematical creativity.

### 2.5 The relation between deep learning approach and psychological resilience

Psychological resilience is typically defined as the ability to bounce back or cope successfully despite substantial adversity (Abate et al., 2024). Psychological resilience brings positive improvement or enhanced adaptive outcomes (Becoña Iglesias, 2006), enabling individuals to withstand significant adversity. Fan et al.’s research on Chinese university students’ mental health conditions adopted the Psychological Resilience Dynamic System Model. This model emphasizes that psychological resilience is a dynamic system whose development is predicted by the integration of individual internal capabilities and external support factors (Fan et al., 2024). This means psychological resilience is not static but a process that individuals continuously improve as their environment changes. Interestingly, deep learners tend to view failure as learning opportunities, which highly aligns with the core characteristic of “recovering from adversity” in psychological resilience (Carmeli and Hartmann, 2024). Researchers have further found that deep learning has a negative correlation with emotional exhaustion (Hu and Yeo, 2020). In this context, psychological resilience can be interpreted as the ability to initiate, persist, and engage in learning when feeling emotionally low during the learning process (Kayes et al., 2024). Therefore, the higher the tendency toward deep learning in the learning process, the more it may reduce the negative effects of emotional exhaustion, thereby enhancing individual psychological resilience. Therefore, we propose hypothesis H2: Deep learning approach positively predicts psychological resilience.

### 2.6 The relation between psychological resilience and mathematical creativity

According to componential theory of creativity, creativity is not the product of a single factor but the result of multiple interconnected components working together. These core components include domain-relevant skills, creativity-relevant processes, and task motivation (Amabile, 1983). Psychological resilience, as an important positive psychological capital, can stimulate intrinsic motivation and enhance creativity (Riaz et al., 2023). Multiple studies indicate that psychological resilience can positively predict and promote creativity. Research has found strong associations between psychological resilience and cognitive creativity, social creativity, as well as its mediating role between negative emotions and creativity (Li et al., 2022; Zeng et al., 2022). In enterprise and organizational environments, psychological resilience is considered one of the key volitional factors for enhancing employee creativity (Mualimin et al., 2025). The promoting effect of psychological resilience on these types of creativity may be equivalent to mathematical creativity. For example, psychological resilience may regulate negative emotions when mathematical innovative thinking is blocked, thereby stimulating mathematical creativity. Therefore, the present study proposes hypothesis H3: Psychological resilience can also positively predict mathematical creativity from a psychological perspective.

### 2.7 The theoretical and conceptual basis of the hypotheses

We established our theoretical framework based on three influential theories of creativity. The first is The Systems Model of Creativity, proposed by Mihaly Csikszentmihalyi, which conceptualizes creativity as the result of the dynamic interaction among the individual, the field, and the domain, rather than as a mere product of personal traits (Ali, 2018). The individual component encompasses factors such as personality traits, cognitive abilities, motivation, domain-relevant skills, and demographic background variables (Csikszentmihalyi and Lebuda, 2017). In this study, we included age and gender as part of this dimension. The domain refers to specific systems of knowledge, symbols, rules, and practices—such as science, art, or mathematics. Within this dimension, we incorporated math score in college entrance examination as a variable.

The domain dimension is also embedded within the Componential theory of creativity, which posits that creativity is not a single trait but the result of interactions among three major components: domain-relevant skills, creativity-relevant processes, and task motivation. In terms of task motivation, we incorporated a deep learning approach and creative self-efficacy as variables (Amin et al., 2023).

In addition, we integrated Self-regulation theory, a framework that focuses on how individuals guide their developmental processes, select and pursue goals, and adjust goal-directed behaviors according to personal and environmental opportunities and constraints (Newman and Newman, 2020). Psychological resilience, as a key factor in adjusting mindset and pursuing goals when facing adversity, is considered a variable under this theory.

**Figure 1:**
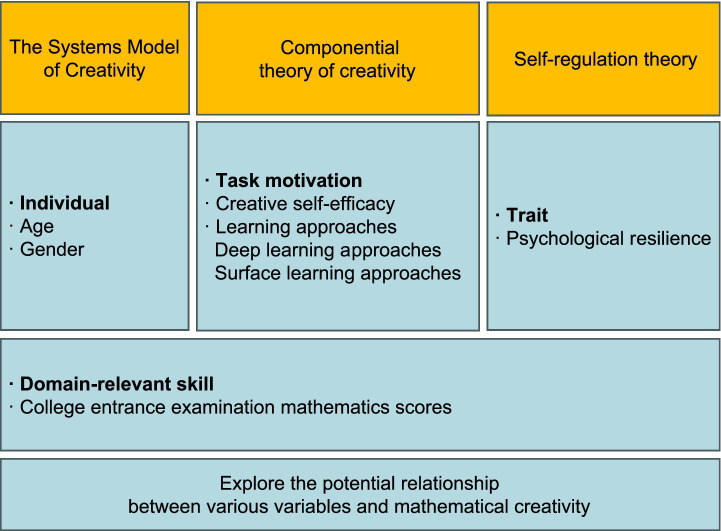
Theoretical model built on the conceptual basis of each variable.

In summary, these theories jointly informed the selection of key variables in this study, and the conceptual framework constructed on this basis is illustrated in Figure 1.

### 2.8 The method integrating structural equation modeling and network analysis

Structural Equation Modeling is a statistical method developed by Joreskog by integrating factor analysis (Zhang et al., 2020), regression analysis, and path analysis techniques, aimed at testing and estimating causal relationships among complex variables, particularly latent variables (Fassinger, 1987), thereby providing more rigorous and accurate quantitative analysis tools for research in social sciences and other fields. Using it, we can accurately quantify and test the relationships among learning approaches, psychological resilience, and mathematical creativity (Yuan and Bentler, 2006), having stronger predictive power compared to traditional path analysis and multiple regression (Hidayat and Wulandari, 2022).

Cross-sectional network analysis is a method based on graph theory and statistics, primarily used to study the relational structures and interactions among elements in complex systems at specific time points (Borsboom et al., 2021). This method represents entities in the system as nodes and their connections as edges, thereby constructing network models that reveal structural characteristics, key elements, and functional patterns within the system, widely applied in psychological research (Briganti et al., 2024). The intuitive network structure representation of all nodes and edges helps identify the group structure of deep learning approach, psychological resilience, mathematical creativity, and their control variables, finding the most central network nodes.

Using structural equation modeling reveals overall hypothetical models among variables, but theoretically presupposed models may be distorted, while using cross-sectional network analysis supplements details, revealing more fine-grained and complex node centrality relationships within or among these latent variables, though it’s difficult to infer causal relationships. The present study will integrate these two methods for complementary advantages, with network analysis supplementing consideration of partial correlation relationships among multiple control variables and three core variables. If this mediating relationship still exists under such more realistic and complex network relationships, it proves that our constructed mediation model is sufficiently stable, thereby enhancing the scientific validity and reliability of this study’s conclusions (Du et al., 2025).

### 2.9 Current study

In summary, existing research has separately proven strong correlations among learning approaches, psychological resilience, and creativity, and has verified the close association between emotions and learning approach choices. However, research on the mechanisms by which learning approaches and psychological resilience, respectively, activate mathematical creativity remains lacking, and no research has appeared on psychological resilience mediating learning approaches and mathematical creativity.

Based on this, the present study will comprehensively employ structural equation modeling and cross-sectional network analysis methods to explore whether deep learning approach can positively predict mathematical creativity, whether psychological resilience serves as a mediating variable, and explore key network nodes affecting mathematical creativity. This study aims to deeply investigate the relationships among deep learning approach, psychological resilience, and mathematical creativity by establishing structural equation models among the three to explain their dynamic associations. Additionally, we will adopt cross-sectional network analysis to intuitively display the network relationships among the three and identify key nodes in the network, thereby providing reliable support for the cultivation, enhancement, and predictive analysis of university students’ mathematical creativity (Zhang Z. et al., 2025).

## 3 Method

### 3.1 Participants and procedures

This study employed a hybrid online-offline sampling approach to collect data from college students enrolled in Chinese universities. The offline questionnaire was conducted through convenience sampling across universities in Guangdong Province (Zhang L. et al., 2025), while the online questionnaire was distributed via the professional platform through SoJump.com (wjx.cn) and disseminated through snowball sampling (Leighton et al., 2021), utilizing social media platforms such as WeChat and QQ for large-scale distribution (Mei and Brown, 2018). The advantage of this design lies in the fact that the seed respondents for the online snowball sampling were partially drawn from participants in the offline convenience sampling—as offline participants originated from diverse geographic regions across China, they served as diversified seed nodes capable of initiating multiple independent referral chains (Kirchherr and Charles, 2018). These participants were invited to recruit their primary and secondary school classmates enrolled in universities of different types and located in various regions throughout China to complete the online questionnaire, thereby effectively promoting geographic dispersion and demographic heterogeneity of the sample (Baltar and Brunet, 2012; Cohen and Arieli, 2011).

To ensure the reliability of online data collection, we implemented the following measures: First, prior to the commencement of the online questionnaire, participants were thoroughly informed about the research objectives, significance, and privacy protection measures, and electronic or written informed consent was obtained, eliminating some participants who did not meet the requirements (Chen et al., 2025). Second, during the questionnaire design phase, the online questionnaire incorporated mandatory response items and logic branching to prevent missing or erroneous responses, while the questionnaire wording was optimized based on a pilot test of the offline questionnaire (*n* = 30) and online questionnaire (*n* = 30), reducing the possibility of participants responding perfunctorily due to poor question formatting (Mei and Brown, 2018). Third, the online questionnaire identified and excluded careless or invalid responses by monitoring response time fluctuations and verifying the internal logical consistency of response options. Data were screened through multiple criteria, including setting a lower threshold for completion time (responses falling below two standard deviations from the mean reading time were deemed invalid), incorporating logic verification items (e.g., consistency checks for reverse-coded items), detecting patterned responding, and removing duplicate samples (based on IP addresses and device identifiers) (Zeng et al., 2025), collecting totaling *N* = 986 valid samples, aged 17–24 years, with an average age of 20.7 years. The sample included 586 female students and 400 male students from multiple universities across China.

This study was approved by the Ethics Committee of South China Normal University. All university students participating in the research did so voluntarily and were presented with informed consent forms before the questionnaire began, confirming voluntary participation before starting to answer. In offline questionnaire completion, university students completed survey questionnaires in batches offline and submitted them anonymously. In online questionnaire completion, university students’ personal information was recorded only with user IDs, with each participant represented by a number instead of their specific name. Finally, through comprehensive questionnaire collection both offline and online, we could deeply understand the interactive relationships among university students’ learning approaches, psychological resilience, and mathematical creativity.

### 3.2 Measures

The survey questionnaire used in this study has five main components: demographic information, learning process questionnaire, psychological resilience scale, mathematical creativity scale, and creative self-short-term scale. Demographic information includes respondents’ gender, age, and math score in college entrance examination, with math score in college entrance examination serving as an indicator of mathematical ability. To adapt scales developed for countries outside China, all scales we used have been validated as Chinese scales with good reliability and validity. To make research results comparable between countries and regions, data inequality issues were addressed before collection.

#### 3.2.1 Learning process questionnaire

The measurement of learning approaches used the revised two-factor study process questionnaire (R-SPQ-2F) developed by Biggs et al. (2001), sinicized by Wang et al. (2021), and structurally studied by Mu (2009). It consists of 20 items, including two factors: deep approach and surface approach, with 10 questions for each learning approach, four subscales of deep motivation and strategy, surface motivation and strategy, each with 5 items, using 5-point scoring, where 1 = “very inconsistent” and 5 = “very consistent.” In this study, when scoring, surface motivation was reverse scored and added to deep motivation scores to obtain the total score. Higher total scores indicate greater tendency toward deep learning. The Cronbach’s alpha of this scale ranged from 0.539 to 0.767.

#### 3.2.2 Psychological resilience scale

This study used the CD-RISC Chinese version (China version of the Connor-Davidson resilience scale) translated and adapted by Zhang and Yu to assess university students’ psychological resilience levels (Yu and Zhang, 2007). The scale consists of 25 items, divided into three dimensions: tenacity, strength, and optimism. Tenacity includes 13 items (items 11–23), strength includes 8 items (items 1, 5, 7, 8, 9, 10, 24, 25), and optimism includes 4 items (items 2, 3, 4, 6). Using 5-point scoring, where 1 = “never” and 5 = “always like this,” higher scores indicate higher levels of psychological resilience. The Cronbach’s alpha of this scale was 0.91.

#### 3.2.3 Mathematical creativity scale

This study used the mathematical creativity scale compiled by Wei (2023). Each question on the scale uses first-person description to describe a mathematical situation or attitudinal tendency. It is divided into four dimensions: awareness of exploring mathematical problems (referring to students having strong curiosity about mathematics, enjoying discovering and proposing mathematical problems), ability to explore mathematical problems (referring to students having the ability to solve general mathematical problems), ability to solve non-routine problems (referring to students being able to use divergent thinking when facing non-routine mathematical problems like open mathematical problems, proposing different solution approaches and plans), and forming mathematical thinking (referring to students being able to use mathematical thinking to view and handle some real-life problems). Each dimension has 5 items, using 5-point scoring, where 1 = “very inconsistent” and 5 = “very consistent.” The Cronbach’s alpha of this scale was 0.93.

#### 3.2.4 Creative self short-term scale

This study was selected from the Short Scale of Creative Self (SSCS) (Karwowski et al., 2018). SSCS consists of 11 items, of which we only selected 6 items measuring creative self-efficacy, for example: “I clearly know that I have the ability to effectively solve complex problems.” Using 5-point scoring, where 1 = “very inconsistent” and 5 = “very consistent.” The Cronbach’s alpha of this scale was 0.81.

All measurement tools will be displayed in full in the Appendix at the end of this article.

## 4 Data analysis

This study used SPSS 26.0 software for structural equation model processing, including mediation model verification with control variables, descriptive analysis, independent sample t-tests, etc. Mediation models with control variables can use control variables to eliminate confounding bias, ensuring the purity of mediation relationships (Adzrago and Williams, 2023). Descriptive statistics are used to describe basic characteristics of data, providing simple summaries about samples and measurements (Mishra et al., 2019). Independent sample t-tests are commonly used hypothesis testing methods in statistics, mainly used to compare whether there are significant differences between the means of two independent sample groups (Chatzi, 2025).

This study also used R studio software for cross-sectional network analysis model processing, including network structure estimation, network inference analysis, confidence intervals, difference testing, and stability testing. Network structure estimation aims to infer conditional dependency relationships (edges) among variables (nodes) from data collected at a single time point (Burger et al., 2023). Network inference analysis includes strength, closeness, and expected influence. Strength reflects the degree of connection tightness or information exchange activity between a node and its surrounding environment; nodes with high closeness centrality can spread information to the entire network more quickly or receive information from other nodes more quickly; nodes with high expected influence have stronger potential prediction on other nodes overall (Qiu et al., 2024). Confidence intervals (CI) quantify uncertainty by calculating confidence intervals of network parameters in bootstrap samples (Sharma and Yadav, 2024). Difference testing examines edge weight representing the strength or frequency of connections between two nodes in the network (Cai et al., 2022). Stability testing can ensure that identified core nodes (nodes with high centrality) maintain their importance under different samples (Sun H. L. et al., 2024), using CS-C coefficients to reveal network connections or nodes that remain robust under data perturbation and have important functions. Its value should be at least greater than 0.25, preferably greater than 0.5 (Epskamp, 2020).

## 5 Results

### 5.1 Descriptive analysis

The descriptive statistical analysis results of the mean and standard deviation for math score in college entrance examination, psychological resilience, mathematical creativity, age, creative self-efficacy, learning approaches, and gender are shown in Table 1.

**Table 1:** Descriptive analysis.

Labels	*N*	*M*	SD
Math score in college entrance examination	986	107.2	18.897
Psychological resilience	986	97.8	10.872
Mathematical creativity	986	95.1	20.626
Age	986	20.7	1.130
Creative self-efficacy	986	20.6	4.659
Learning approaches	986	13.9	10.835
Gender	986	0.4	0.491

### 5.2 Correlation analysis

**Figure 2:**
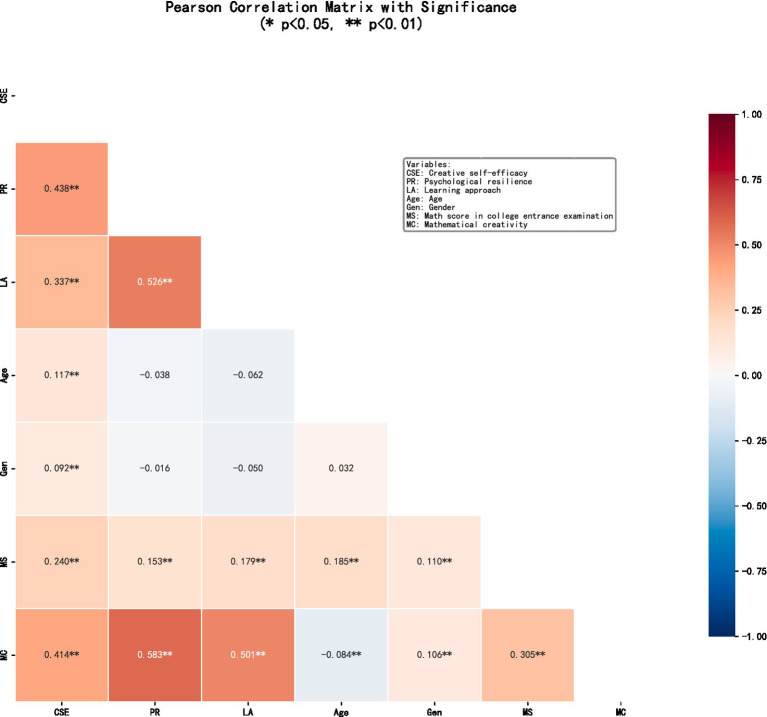
Pearson correlation matrix among the study variables.

A Pearson correlation analysis was conducted to examine the relationships various variables, and mathematical creativity. The results showed significant positive correlations among the main variables (*p* < 0.01). Creative self-efficacy, learning approaches, and psychological resilience were strongly and positively associated with mathematical creativity, indicating that students with higher self-efficacy and deeper learning approach tend to demonstrate greater creativity in mathematics. More specific details are shown in Figure 2.

### 5.3 Tests for mediating effects

Table 2 presents the results of a mediation analysis examining whether psychological resilience mediates the relationship between learning approaches and mathematical creativity outcomes, controlling the variables of math score in college entrance examination, age, gender, and creative self-efficacy, which can eliminate potential confounding effects, improve estimation accuracy, enhance the robustness of results, and reflect theoretical rigor. Among the control variables, math score in college entrance examination (*β* = 0.013, *p* = 0.862) and age (*β* = −0.090, *p* = 0.248) were nonsignificant, while gender (*β* = 0.112, *p* < 0.001) and creative self-efficacy (*β* = 0.163, *p* < 0.001) showed significant direct effects on mathematical creativity. The analysis includes three models: direct effects on mathematical creativity, effects on the mediator (psychological resilience), and the full mediation model. Significant coefficients are marked with asterisks (*p < 0.05, **p < 0.01), with standardized beta coefficients, standard errors, t-values, and *p*-values reported for each pathway.

**Table 2:** Mediation analysis results (*N* = 986).

	Mathematical creativity			Psychological resilience					Mathematical creativity						
	B	Standard Error	*t*	*p*	*β*	B	Standard Error	*t*	*p*	*β*	B	Standard Error	*t*	*p*	*β*
Constant	102.953**	27.145	3.793	0.000	-	95.117**	14.109	6.741	0.000	-	34.126	25.741	1.326	0.185	-
Math score in college entrance examination	0.668	1.693	0.395	0.693	0.033	0.547	0.880	0.621	0.535	0.051	0.273	1.569	0.174	0.862	0.013
Age	−2.304	1.527	−1.508	0.132	−0.126	−0.922	0.794	−1.161	0.246	−0.096	−1.637	1.417	−1.155	0.248	−0.090
Gender	4.368**	1.104	3.956	0.000	0.104	−0.441	0.574	−0.768	0.443	−0.020	4.687**	1.024	4.577	0.000	0.112
Creative Self-efficacy	1.235**	0.124	9.924	0.000	0.279	0.711**	0.065	10.991	0.000	0.305	0.720**	0.122	5.893	0.000	0.163
Learning Approaches	0.773**	0.053	14.569	0.000	0.406	0.420**	0.028	15.242	0.000	0.419	0.469**	0.055	8.570	0.000	0.246
Psychological Resilience											0.724**	0.057	12.701	0.000	0.381
*R* ^2^	0.338			0.356					0.432						
Adjusted *R*^2^	0.334			0.353					0.428						
*F*	*F* (5,980) = 99.998, *p* = 0.000			*F* (5,980) = 108.375, *p* = 0.000					*F* (6,979) = 123.851, *p* = 0.000						

*p < 0.05 **p < 0.01, male = 1, female = 0.

The results of the structural equation model showed that deep learning approach and psychological resilience had significant positive effects on mathematical creativity, and creative self-efficacy as a control variable was also significantly related to mathematical creativity.

Table 3 presents bootstrapped mediation analysis results testing psychological resilience as a mediator between learning approaches and mathematical creativity. The analysis includes path coefficients for the indirect effect (a*b), individual paths (a: X → M, b: M → Y), direct effect (c’), and total effect (c). The 95% confidence intervals and significance tests confirm partial mediation, indicating that psychological resilience significantly mediates the relationship while a direct effect also remains significant.

**Table 3:** Tests for mediating effects.

Path	Sign	Meaning	Effect Value	95% CI		Standard Error SE value	z value/t value	*p* value	Conclusion
				Lower	Upper				
Learning Approaches= > Psychological Resilience= > Mathematical Creativity	a*b	Indirect Effect	0.304	0.115	0.201	0.022	13.885	0.000	Partial Mediation
Learning Approaches= > Psychological Resilience	a	X= > M	0.420	0.366	0.474	0.028	15.242	0.000	
Psychological Resilience= > Mathematical Creativity	b	M= > Y	0.724	0.612	0.835	0.057	12.701	0.000	
Learning Approaches= > Mathematical Creativity	c’	Direct Effect	0.469	0.362	0.576	0.055	8.570	0.000	
Learning Approaches= > Mathematical Creativity	c	Total Effect	0.773	0.669	0.877	0.053	14.569	0.000	

The results showed that deep learning approach significantly positively predicted psychological resilience (*β* = 0.420, *t* = 15.242, *p* < 0.001) and the direct effect on mathematical creativity (*β* = 0.469, *t* = 8.570, *p* < 0.001). Psychological resilience also had a significant positive prediction on mathematical creativity (*β* = 0.724, *t* = 12.701, *p* < 0.001). Furthermore, the indirect effect value of deep learning approach predicting mathematical creativity through psychological resilience was 0.304, with a 95% confidence interval [0.201, 0.383] not including 0, and the total effect c = 0.773 including both direct and indirect effects, indicating that psychological resilience plays a partial mediating role between deep learning approach and mathematical creativity.

**Figure 3:**
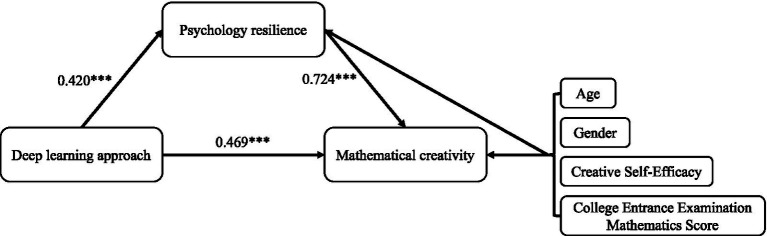
The results of the mediation model.

Figure 3 illustrates the mediation model where psychological resilience mediates the relationship between deep learning approach and mathematical creativity. Path coefficients show the direct effect from deep learning approach to mathematical creativity (0.469), the effect through psychological resilience (0.420 → 0.724), demonstrating partial mediation. Control variables include age, gender, creative self-efficacy, and math score in college entrance examination. All displayed paths are statistically significant.

### 5.4 Tests for network analysis

**Figure 4:**
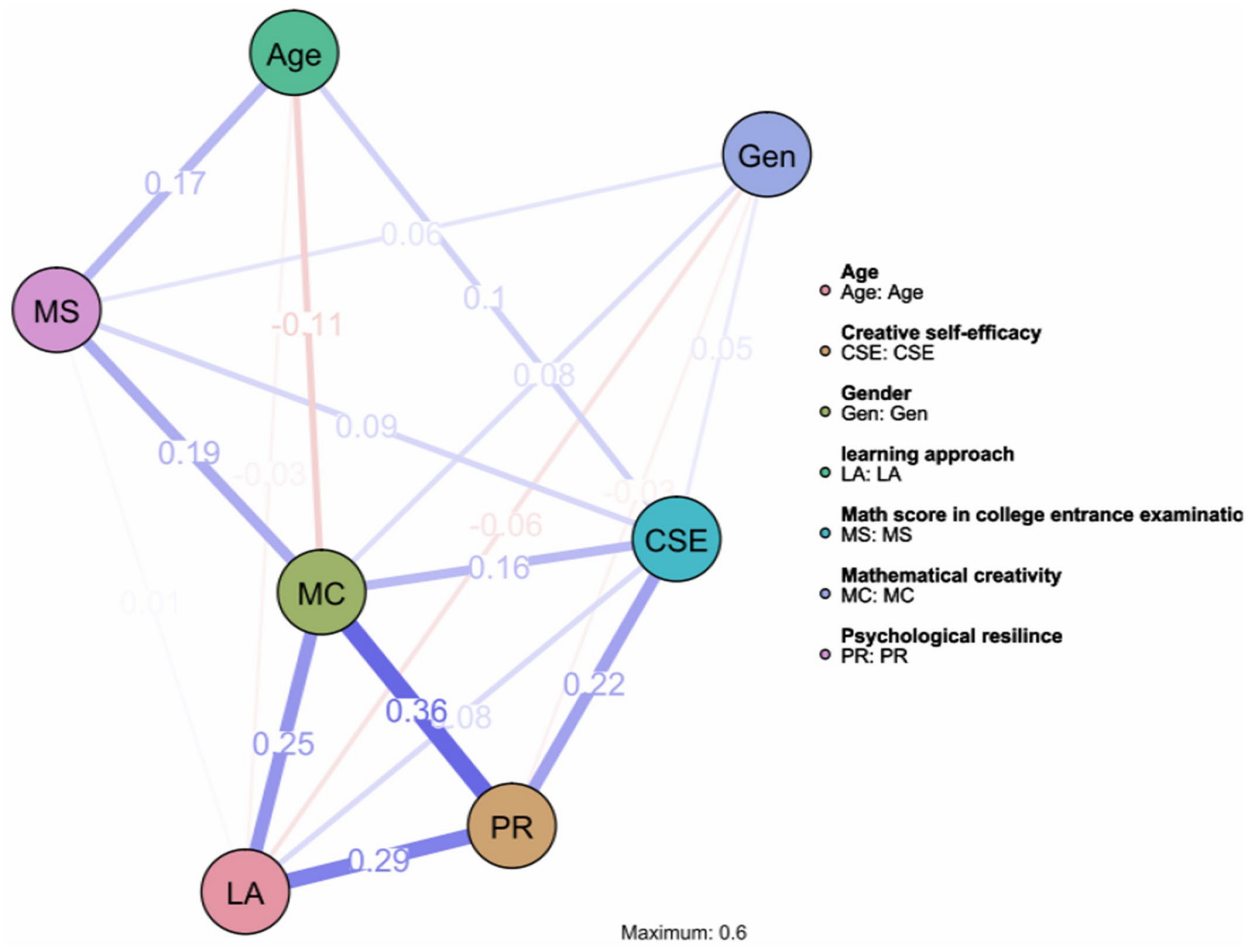
The results of network analysis.

This network diagram displays the interconnections among study variables using network analysis (see Figure 4). Node colors represent different variable types: age (green), gender (blue), creative self-efficacy/CSE (teal), mathematical creativity/MC (dark green), learning approach/LA (pink), psychological resilience/PR (orange), and math score in college entrance examination /MS (purple). Edge thickness and saturation indicate association strength, with thicker lines representing stronger relationships. The network reveals the complex interdependencies among psychological and demographic variables influencing mathematical creativity.

We drew a cross-sectional network analysis diagram. In the network structure estimation diagram, nodes represent different variables, including age, core self-evaluation (CSE), learning approaches (LA), mathematical creativity (MC), psychological resilience (PR), gender (Gen), and math score in college entrance examination (MS), totaling 7 nodes. The connections between nodes and their numerical values represent the association strength between variables. There are 18 non-zero edges: LA-MC (0.25), LA-PR (0.29), PR-MC (0.36), CSE-PR (0.22), CSE-MC (0.16), CSE-LA (0.08), CSE-Age (0.1), CSE-MS (0.09), CSE-Gen (0.05), Gen-MC (0.08), Gen-MS (0.06), Age-MS (0.17), MS-MC (0.19), MS-LA (0.01) showing positive correlations, while Gen-LA (−0.06), Gen-PR (−0.02), Age-MC (−0.11), Age-LA (−0.03) showed negative correlations. The average weight was 0.105. In this network, the edges with the largest weights were those connecting LA, PR, and MC, which proves that the path of psychological resilience mediating deep learning approach’ prediction on mathematical creativity constructed using structural equation modeling has sufficient stability, providing strong support for this conclusion.

**Figure 5:**
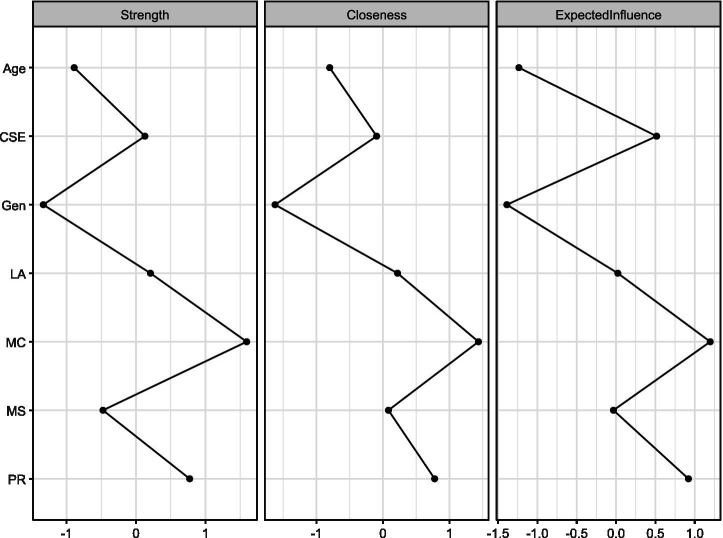
The results of centrality measures for network variables.

Figure 5 displays three key centrality indices for network variables: Strength (sum of absolute edge weights), Closeness (inverse of sum of shortest distances to all other nodes), and Expected Influence (sum of edge weights considering direction). Variables include age, creative self-efficacy (CSE), gender (Gen), learning approach (LA), mathematical creativity (MC), psychological resilience (PR), and mathematics score (MS). The metrics reveal each variable’s relative importance and influence within the network structure.

To study the centrality of each node in the combined network node strength, this study estimated node closeness and betweenness centrality. The above figure is a visualization of these three standardized centrality indices of the network. Regarding node strength, the node with the highest strength was MC (mathematical creativity), while Gen (gender) had the lowest node strength. In terms of node closeness centrality, the node with the highest closeness centrality was still MC. Regarding node expected influence, the highest node was still MC. The overall trends of these three dimensions of node centrality were almost consistent, representing a relatively stable network structure. It is worth noting that psychological resilience ranked second in all three dimensions of node centrality, meaning that psychological resilience plays the most crucial role in the network structure apart from mathematical creativity.

**Figure 6:**
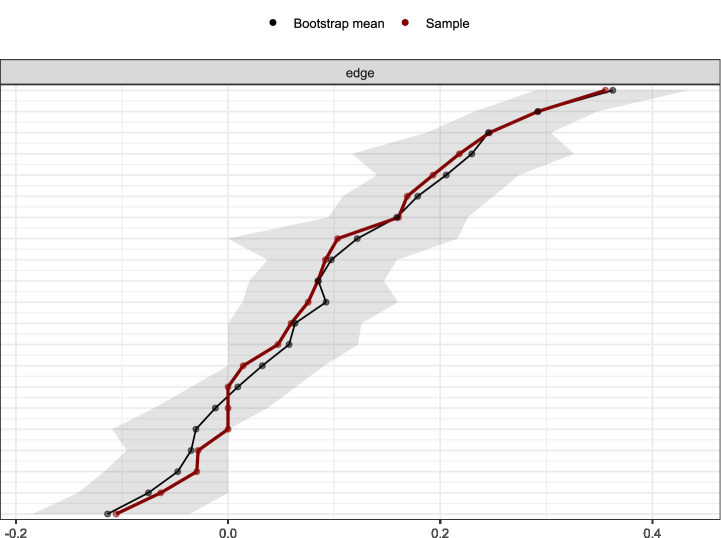
The results of bootstrap stability analysis for edge weights.

**Figure 7:**
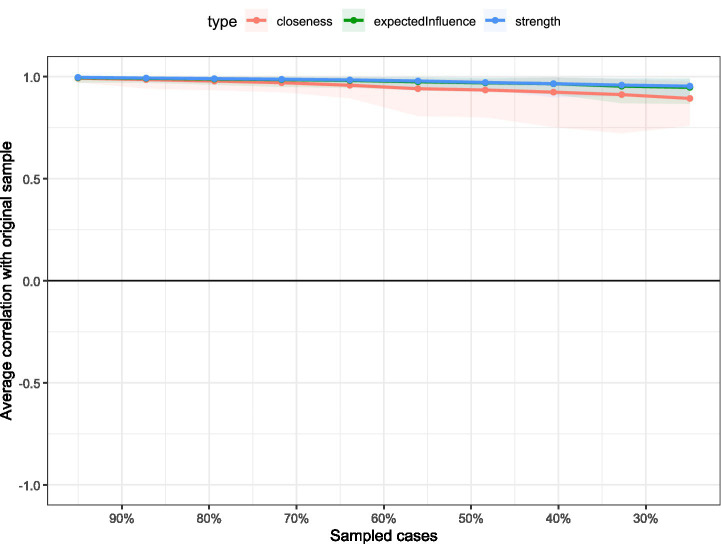
The results of centrality stability analysis across bootstrap samples.

To study network accuracy and stability, 95% CI calculations around edge weights were performed for edge weight accuracy. The visualization of network edge weight accuracy calculation is shown in the Figure 6. The results showed that around most estimated edge weights, the bootstrapped CIs were relatively small, indicating high precision. The visualization of node strength centrality coefficient stability testing is shown in the Figure 7. By evaluating CS-C coefficients to quantify network stability, the results after removing sample sizes showed that all node CS-C coefficients consistently remained above 0.75, indicating very strong network stability.

Figure 6 shows the bootstrap stability analysis for network edge weights. The red line represents the sample edge weights, while the black line shows bootstrap mean values with gray confidence intervals. The close alignment between sample and bootstrap means, along with narrow confidence intervals, demonstrates good stability and reliability of the estimated network structure. The analysis confirms that the observed edge weights are robust and not due to sampling variability.

Figure 7 displays the stability of three centrality measures (closeness, expected influence, and strength) across different bootstrap sample sizes. The y-axis shows average correlation with the original sample, while the x-axis indicates the percentage of sampled cases. High correlations (>0.7) maintained across all sample sizes demonstrate robust stability for all centrality indices, confirming the reliability of centrality rankings in the network analysis.

## 6 Discussion

Based on data from university students across Chinese universities, this study demonstrated that deep learning approach significantly develops mathematical creativity, with psychological resilience playing a partial mediating role.

### 6.1 Validation results of the H1 hypothesis

The findings of this study provide empirical support for Hypothesis 1, demonstrating that deep learning approach significantly and positively predicts mathematical creativity (*β* = 0.469, *p* < 0.001). Students using deep learning approaches actively construct knowledge rather than passively receiving it (Karki and Lamichhane, 2020). In mathematics education, this means students need to face complex real-world problems and use mathematical tools for modeling and solving (Weinhandl and Lavicza, 2021). This learning approach can effectively stimulate students’ curiosity and desire for exploration (Grüning and Krueger, 2024). This ability to abstract and model concrete things can be characterized as abstract thinking ability, the ability to extract general laws and concepts from specific examples (Ai, 2025). Understanding abstract concepts in mathematics such as topology and group theory is precisely the transformation process from surface features to deep abstract features emphasized by deep learning. Therefore, deep learning approaches can enable people to master the essential attributes of mathematical knowledge more skillfully through abstraction and integration of concepts, thereby demonstrating stronger mathematical creativity in mathematical practice.

This finding is like previous research results, where people using deep learning approaches have higher cognitive flexibility (Vedenpää and Lonka, 2014), which develops people’s innovative thinking and creative application abilities (Li et al., 2023), thereby enhancing their creativity. Specifically in the mathematical field, this tendency of deep learning approaches to explore the essential logic of mathematics is closely related to people’s fluency and originality in solving mathematical problems, indeed achieving the purpose of stimulating mathematical creativity (Bicer et al., 2024). Furthermore, Biggers proposed the famous Presage-Process-Product (PPP) model for student learning conditions (Liu et al., 2015). This theory suggests that deep learning approaches typically lead to higher quality learning outcomes and deeper understanding, while surface learning approaches may lead to superficial mastery of knowledge and lower academic performance. This study expanded the “product” part of this theory, deeply explaining the role that deep learning approaches play in creativity development, proving that university students using deep learning approaches are more conducive to enhancing their mathematical creativity.

However, previous research on this topic mainly focused on the relationship between deep learning approaches and mathematical academic performance (Everaert et al., 2017; Zhao et al., 2022), and research has proven that the correlation between mathematical creativity and mathematical achievement in adults is not as significant as in children (Chan, 2016; Meier et al., 2021). Because age groups differ, deep learning approaches may not necessarily have similar strong correlations with mathematical creativity. This study further confirmed that this conclusion also applies to university students’ mathematical creativity.

This enlightens us to actively develop students’ deep learning approaches through inquiry-based learning and personalized teaching in educational instruction, which is more conducive to stimulating students’ mathematical creativity and promoting students’ creativity development in higher education.

Conversely, our results implicitly highlight the limitations of surface learning approach. Students who rely primarily on memorization and superficial processing are likely to develop fragmented knowledge structures that constrain their ability to engage in the divergent thinking and knowledge reconstruction required for mathematical creativity (Rahmatin et al., 2025; Roehe et al., 2024). This interpretation is consistent with previous research demonstrating that surface learning approach is associated with rigid thinking patterns and limited problem-solving flexibility (Li et al., 2025).

### 6.2 Validation results of the H2 hypothesis

This study also confirmed the H2 hypothesis that deep learning approach can positively promote the development of psychological resilience. This is because deep learners can develop stronger problem-solving abilities (Lee and Hur, 2024), making them more confident and capable of overcoming difficulties when facing challenges, thereby enhancing self-efficacy, and high self-efficacy can predict better mental health and coping abilities (Qin et al., 2023). At the same time, psychological resilience includes not only responding to adversity itself but also the ability to grow from adversity, which coincides with the growth mindset of deep learning, encouraging individuals to continuously explore, reflect, and transcend (Damanik and Muhammad, 2025). Individuals with deep learning orientations are more inclined to view challenges as opportunities for learning and growth rather than insurmountable obstacles. This positive attribution style and adaptive response is precisely the core manifestation of psychological resilience.

This finding is like previous research, namely that deep learning approach has negative correlations with emotional exhaustion, meaning that people adopting deep learning approach has lower possibilities of emotional exhaustion and thus higher psychological resilience (Hu and Yeo, 2020). At the same time, deep learners demonstrate stronger endurance than others when facing problems, enjoying the process of finding essential laws of things in energy-consuming thinking, which coincides with the trait of maintaining optimistic attitudes while in adversity (Carmeli and Hartmann, 2024). This tenacity is also one of the key dimensions measured by psychological resilience. Furthermore, deep learning approach understanding the essence, establishing knowledge connections, and pursuing meaning-oriented learning (Koopman et al., 2014). This encourages students to proactively set long-term goals and view difficulties as part of the learning process. They maintain motivation by redefining failure and seeking meaning, which reflects the goal maintenance mechanism in self-regulation theory and is a core psychological component of psychological resilience (Wang, 2025).

### 6.3 Validation results of the H3 hypothesis

Additionally, the significant positive prediction of mathematical creativity by psychological resilience (*β* = 0.724, *p* < 0.001) confirms Hypothesis 3 and demonstrates the critical role of volitional factors in creative mathematical performance. In the creative process of mathematics, problem-solving is often accompanied by prolonged periods of confusion, logical conflicts, and failures (Riegel, 2021). Therefore, psychological resilience, as a cross-situational self-regulation and recovery mechanism, becomes a key mediator connecting intrinsic motivation and creative cognitive processes (Li B. et al., 2023). Consequently, we propose that psychological resilience can be regarded as a “meta-component” in the component theory of creativity, which regulates an individual’s ability to maintain creative motivation and flexible thinking under long-term cognitive challenges and emotional fluctuations (Liang et al., 2026). This finding responds to componential theory of creativity and is an important extension of this theory in the field of mathematics education. Cognitive creativity, social creativity (Li et al., 2022), and employee creativity (Mualimin et al., 2025) all have strong correlations with psychological resilience, and this is no exception in mathematical creativity.

We further discovered that psychological resilience could predict mathematical creativity more strongly than deep learning approaches. This is because psychological resilience, as a volitional factor, enables students to better cope with obstacles and negative situations during the learning process and transform them into supportive situations, thereby achieving better learning outcomes than expected. It can effectively enhance mathematical problem-solving abilities (Xu et al., 2025) and overcome mathematics-specific anxiety (Johnston-Wilder et al., 2021). As an intrinsic volitional driving force, psychological resilience prompts individuals not to retreat when facing difficulties and to continue investing time and energy, which is particularly important for creative activities that require long-term investment and exploration (Amin et al., 2023). While deep learning approaches also require investment, they focus on optimizing cognitive processes rather than overcoming external or internal obstacles. In cognitive psychology, psychological resilience and creativity share brain functional networks (Sun J. et al., 2024), meaning psychological resilience is not only a behavioral manifestation but is deeply rooted in neurocognitive mechanisms, providing intrinsic stability and support for creative thinking.

Therefore, this study believes that psychological resilience serves as a mediating variable in the mediation effect chain between learning approaches and mathematical creativity. In previous research, studies involving psychological resilience and concepts in the field of mathematics, like mathematical creativity, only proved their correlational relationships. At the same time, psychological resilience has been researched as a mediating variable in creativity and other related factors (Xu et al., 2022). This finding is an important extension to this field.

### 6.4 About network analysis

In terms of network node centrality, mathematical creativity was strongest in all three dimensions, indicating that mathematical creativity is not a passive result but can conversely predict and integrate other related variables, such as improving learning approach efficiency, promoting deepening of mathematical abilities, and even enhancing individual psychological resilience when facing mathematical challenges (Cropley et al., 2017; Meier et al., 2024). This confirms that mathematical creativity holds an important position in the educational field.

The second strongest node centrality in the network was psychological resilience. Since creative activities themselves are accompanied by uncertainty and risk of failure (Beaulieu, 2022), when facing setbacks or negative feedback in mathematical creativity activities, higher psychological resilience may help people better handle these emotions, maintain positive attitudes, and continue trying (Afek et al., 2021). Therefore, it presents a central connection with mathematical creativity in the network.

Convergent evidence from structural equation modeling and network analysis provides robust validation of our theoretical framework. The hypothesized mediation pathway identified through theory-driven SEM emerged as the strongest connection in data-driven network analysis, demonstrating genuine psychological phenomena rather than priori assumptions.

### 6.5 Limitations and outlook

This study also has some limitations, hoping for future improvements. First, the scope of mediating variable selection is limited, and there may be omitted variable problems. Although this study selected representative psychological resilience as a mediating variable, due to research constraints, the questionnaire design could not cover other potential mediating factors, which may cause bias in path coefficient estimation of existing variables in the model. Future research should conduct broader and deeper literature reviews, adding more possible variables, such as teaching style, peer influence, academic stress, or family background. Additionally, emerging algorithms such as Machine Learning can be adopted for nonlinear relationship exploration to quantify and assess potential impacts of omitted variables. Second, the timeliness of data analysis is insufficient. This study’s network analysis was built on cross-sectional data from a single time point, lacking longitudinal examination of dynamic relationships among variables. For example, in higher education processes, university students’ deep learning approach, psychological resilience, and their action paths on mathematical creativity may change over time. Future research should adopt longitudinal designs, such as Cross-Lagged Panel Analysis, to more accurately clarify causal relationships among variables and changes in network structure. Third, using self-reported questionnaires may lead to bias or inaccuracies due to social desirability or subjective perceptions. Future research should incorporate multi-source or behavioral measures to obtain more objective and reliable data. Forth, since all participants were Chinese university students, the results may not fully apply to other cultural or educational settings, suggesting that future research should include participants from diverse cultural backgrounds to enhance the generalizability of the findings. Finally, the depth of discussion on action mechanisms needs expansion. Although this study revealed the existence of psychological resilience’s mediating effect in the process of learning approaches influencing mathematical creativity, deeper theoretical models and association mechanisms for this effect remain to be explored and explained in future research. Future research should appropriately combine qualitative research, such as interviews or case studies, to understand more carefully how psychological resilience specifically helps students overcome difficulties and cope with setbacks during deep learning processes, thereby stimulating mathematical creativity.

## 7 Conclusion

This study focused on exploring the correlational relationships among deep learning approach, psychological resilience, and mathematical creativity, comprehensively employing structural equation modeling and cross-sectional network analysis methods, revealing that deep learning approach positively predicts mathematical creativity, with psychological resilience as a mediating variable, and that enhancing psychological resilience is a relatively effective way to stimulate mathematical creativity. These findings help researchers truly understand the deep internal associations among them.

## Data Availability

The original contributions presented in the study are included in the article/Supplementary material, further inquiries can be directed to the corresponding authors.
