# Supplementary material for: The mediating role of psychological resilience in the relationship between deep learning approach and mathematical creativity: integrating structural equation model and network analysis
Source: Front Psychol. 2025 Nov 27;16:1697817. doi: 10.3389/fpsyg.2025.1697817 (PMC12695773; doi:10.3389/fpsyg.2025.1697817)
Supplement: Supplementary file 1 [file Supplementary_file_1.pdf]

Dear Student,

Welcome! This questionnaire is mainly designed to understand the relationship between your mathematical creativity and certain non-intellectual factors. There are no right or wrong answers, so please answer based on your real experiences and personal feelings. All the information you provide will be used for academic purposes only and has nothing to do with your teacher or your grades. We will keep your information confidential, so please feel free to complete it. Thank you for your participation!

School：________________ Grade：_________ Age：_________ Gender：_______

Math score in college entrance examination：______

**Part one: The following is a description about learning approach. Read it and respond accordingly by marking "√" on the number that best reflects your actual situation.**		**very inconsistent**	**inconsistent**	**unclear**	**consistent**	**very consistent**
1	Studying sometimes gives me a strong sense of personal satisfaction.	1	2	3	4	5
2	I like spending extra time researching problems until I find a satisfactory answer.	1	2	3	4	5
3	My goal is just to pass exams, so I want to spend as little effort as possible on studying.	1	2	3	4	5
4	I only study seriously the content emphasized in class and the key points in the textbook.	1	2	3	4	5
5	I feel that any problem can become interesting as long as I put in the effort.	1	2	3	4	5
6	I find most new topics fascinating and often spend extra time to gather more related information.	1	2	3	4	5
7	I am not interested in the courses I study, so I spend as little effort as possible.	1	2	3	4	5
8	Sometimes I learn by rote memorization because it helps me firmly remember some knowledge I don't understand.	1	2	3	4	5
9	I feel that researching academic problems can be as exciting as reading a good novel or watching a good movie.	1	2	3	4	5
10	For some important issues, I ponder repeatedly until I thoroughly understand them.	1	2	3	4	5
11	I've found that memorizing the key parts is	1	2	3	4	5

	enough to pass most exams, and there is no need to fully understand them.					
12	I usually only study what the teacher requires and don't spend effort on anything else.	1	2	3	4	5
13	I study hard because I find the study material very interesting.	1	2	3	4	5
14	If I am interested in the topics discussed in class, I will use my spare time to learn more about them.	1	2	3	4	5
15	When you only need a basic understanding of a problem, in-depth study is meaningless; it only causes confusion and wastes time.	1	2	3	4	5
16	I believe teachers also don't want students to spend a lot of time studying material that won't be on the exam.	1	2	3	4	5
17	For most courses, I go to class with questions in mind and hope to get answers during the lesson.	1	2	3	4	5
18	I believe it is important to read extracurricular materials related to the course.	1	2	3	4	5
19	I think content that won't appear on the exam is not important.	1	2	3	4	5
20	I believe the best way to pass exams is to memorize the answers to questions that might be on them.	1	2	3	4	5

**Part two: The following is a description about psychological resilience. Read it and respond accordingly by marking "√" on the number that best reflects your actual situation.**		**never**	**seldom**	**sometimes**	**often**	**always**
1	I can adapt to changes (in my environment and different situations).	1	2	3	4	5
2	I have some close and secure relationships (such as family, close friends, and romantic relationships).	1	2	3	4	5
3	When I encounter problems I cannot solve, sometimes fate or chance can help.	1	2	3	4	5
4	No matter what happens in my life, I can handle it well.	1	2	3	4	5
5	Past successes give me confidence to face new challenges.	1	2	3	4	5

6	When facing situations, I can always see the humorous or interesting side of things.	1	2	3	4	5
7	After experiencing pressure and challenges, I have become stronger than before.	1	2	3	4	5
8	After illness, injury, or suffering, I can quickly adjust and recover.	1	2	3	4	5
9	Whether things are good or bad, I believe there is a reason behind them.	1	2	3	4	5
10	No matter the outcome, I will do my best.	1	2	3	4	5
11	Even with obstacles, I believe I can achieve my goals.	1	2	3	4	5
12	Even when things seem hopeless, I do not give up easily.	1	2	3	4	5
13	When pressure or crises arise, I know where to seek help.	1	2	3	4	5
14	Under pressure, I can still think deeply and focus on the problem.	1	2	3	4	5
15	I like to take the lead in solving problems rather than letting others take control.	1	2	3	4	5
16	I do not get discouraged by failure.	1	2	3	4	5
17	When dealing with life's challenges and difficulties, I think I am a strong person.	1	2	3	4	5
18	When necessary, I can make unconventional or difficult decisions instead of going with the flow.	1	2	3	4	5
19	I can handle unpleasant or painful emotions (such as sadness, fear, and anger).	1	2	3	4	5
20	When dealing with life’s problems, I have to act on intuition.	1	2	3	4	5
21	In life, I have clear goals and a sense of direction.	1	2	3	4	5
22	I feel that I can take control of my life rather than being driven by external circumstances.	1	2	3	4	5
23	I like challenges.	1	2	3	4	5
24	No matter what obstacles I encounter in life, I will work hard to achieve my goals.	1	2	3	4	5
25	I feel proud of my achievements.	1	2	3	4	5

Part three: The following is a description about mathematical creativity. Read it and respond accordingly by marking "√" on the number that best reflects your actual situation.		very inconsistent	inconsistent	unclear	consistent	very consistent
1	I enjoy discovering and posing mathematical problems.	1	2	3	4	5
2	I like to think and have a strong desire for knowledge.	1	2	3	4	5
3	I often pursue challenging mathematical problems.	1	2	3	4	5
4	I enjoy doing math problems and the pleasure of solving them.	1	2	3	4	5
5	I like doing unconventional and interesting math puzzles.	1	2	3	4	5
6	When I encounter very difficult math problems, I always try every possible way to solve them.	1	2	3	4	5
7	I can infer the mathematical concepts and theorems from the problems/activities that are taught in textbooks.	1	2	3	4	5
8	For some math problems, I can see the solution right away just by looking at the problem.	1	2	3	4	5
9	I often analyze, reflect on, and summarize my problem-solving approaches.	1	2	3	4	5
10	I often have different opinions on math problems that classmates generally agree on.	1	2	3	4	5
11	For incorrect math problems, I can always think of ways to prove their errors.	1	2	3	4	5
12	I often transform unfamiliar math problems into forms I am familiar with.	1	2	3	4	5
13	I am able to apply existing problem-solving methods to new math problems, thereby forming my own ideas.	1	2	3	4	5
14	I often understand math problems from different angles.	1	2	3	4	5
15	When solving math problems, I often have several methods of solving them.	1	2	3	4	5
16	For general math problems, I also often have unique solutions.	1	2	3	4	5
17	I can consider math problems comprehensively and analyze all possible	1	2	3	4	5

	situations.					
18	I am very familiar with open-ended math problems and have good solutions or ideas for such problems.	1	2	3	4	5
19	I always notice hidden conditions in math problems and come up with new questions.	1	2	3	4	5
20	For categorized problems, I can always think of many ways to categorize them.	1	2	3	4	5
21	For problems with uncertain conclusions, I can always find several different conclusions.	1	2	3	4	5
22	I often think about whether the teacher's teaching methods suit me.	1	2	3	4	5
23	I often reflect on my learning methods and adjust them when necessary.	1	2	3	4	5
24	I believe mathematics plays a very important role in life.	1	2	3	4	5
25	When reading extracurricular books, if I find many numbers in them, I always like to figure out what these numbers mean or their patterns.	1	2	3	4	5
26	In life, when I encounter situations related to mathematics, I instinctively use mathematical methods to solve them.	1	2	3	4	5

**Part four: The following is a description about creative self-efficacy. Read it and respond accordingly by marking "√" on the number that best reflects your actual situation.**		**very inconsistent**	**inconsistent**	**unclear**	**consistent**	**very consistent**
1	l know l can efficiently solve even complicated problems.	1	2	3	4	5
2	l trust my creative abilities.	1	2	3	4	5
3	Compared to my friends, l am distinguished by my imagination and ingenuity.	1	2	3	4	5
4	Many times l have proven that I can cope with difficult situation.	1	2	3	4	5
5	l am sure l can deal with problems requiring creative thinking.	1	2	3	4	5
6	l am good at proposing original solutions to problems.	1	2	3	4	5
