# Supplementary material for: The mediating role of psychological resilience in the relationship between deep learning approach and mathematical creativity: integrating structural equation model and network analysis
Source: Front Psychol. 2025 Nov 27;16:1697817. doi: 10.3389/fpsyg.2025.1697817 (PMC12695773; doi:10.3389/fpsyg.2025.1697817)
Supplement: Supplementary file 2 [file Supplementary_file_2.pdf]

亲爱的同学：

你好！这份问卷主要用于了解你的数学创造力与部分非智力因素之间的关系。所有答案没有对错之分，请根据你的真实经验和切身感受填写即可。你提供的所有资料仅供学术使用，与你的老师或成绩无关。我们会对你提供的资料保密，请放心填写。谢谢你的参与！

学校：________________ 年级：_________ 年龄：_________ 性别：_______

高考数学成绩：______

一、以下是关于学习定向的描述，看懂即答，请在最符合你实际情况的数字上打“√”。		完全不符	比较不符	不确定	比较符合	完全符合
1	学习有时给我一种很强烈的个人满足感	1	2	3	4	5
2	我喜欢多花一些时间来研究问题，直到找到满意的答案为止	1	2	3	4	5
3	我的目标是考试及格，学习上花的功夫越少越好	1	2	3	4	5
4	我只认真学习课堂上强调的内容及教材中的重点内容	1	2	3	4	5
5	我觉得只要肯投入，任何问题都可以变得很有趣	1	2	3	4	5
6	我觉得大部分新课题都很有趣，常常花额外的时间来获取更多相关信息	1	2	3	4	5
7	我对所学课程不感兴趣，所以就尽可能少花功夫	1	2	3	4	5
8	有时候会通过死记硬背的方式来学习，因为这样可以牢牢的记住一些不理解的知识	1	2	3	4	5
9	我觉得研究学术问题，有时和读一本好的小说或看一部好的电影一样令人兴奋	1	2	3	4	5
10	对一些重要的问题，我反复琢磨，直到能彻底理解	1	2	3	4	5
11	我发现只要把关键部分背熟就能通过大多数考试，并不需要去理解它们	1	2	3	4	5
12	我通常只学老师要求的东西，而不会花精力去学别的	1	2	3	4	5
13	我学习很努力，因为我觉得学习材料非常有趣	1	2	3	4	5
14	如果我对课堂上讨论的问题感兴趣，我就会利用课余时间去了解更多这方面的知识	1	2	3	4	5
15	当你只需对问题略知一二时，深入研究并没什么意	1	2	3	4	5

	义，只能使人困惑和浪费时间					
16	我相信老师也不希望学生花大量的时间去研究那些考试不会考的材料	1	2	3	4	5
17	对于大多数课程，我都是带着问题去上课的，也希望在课堂上能够得到答案	1	2	3	4	5
18	我认为阅读与课程相关的课外材料很重要	1	2	3	4	5
19	我认为考试中不会出现的内容就不重要	1	2	3	4	5
20	我认为通过考试的最好的方法是：把可能考到的问题的答案背下来	1	2	3	4	5

二、以下是关于心理弹性的描述，看懂即答，请在最符合你实际情况的数字上打“√”。		从不	较少	有时	较多	总是
1	我能适应变化（身边环境、不同情况的变化）	1	2	3	4	5
2	我拥有一些亲密、安全的关系（如亲情、挚友、恋情）	1	2	3	4	5
3	当我遇到无法解决的困境时，有时命运或机缘能帮忙	1	2	3	4	5
4	无论我的人生中发生什么事，我都能很好地处理	1	2	3	4	5
5	过去的成功经历，让我有信心面对新的挑战	1	2	3	4	5
6	面对事情时，我总能看到事情幽默、有趣的一面。	1	2	3	4	5
7	历经强压和磨练，我变得比原来更坚强了	1	2	3	4	5
8	在生病、受伤或遭遇苦难后，我很快就可以调整回来	1	2	3	4	5
9	不论事情好坏，我相信事出有因	1	2	3	4	5
10	无论结果怎样，我都会尽自己最大努力去做	1	2	3	4	5
11	纵使有阻碍，我相信我能实现自己的目标	1	2	3	4	5
12	纵使事情看起来没有希望，我仍然不轻言放弃	1	2	3	4	5
13	当压力或危机来临时，我知道可以在哪里获得帮助	1	2	3	4	5
14	在压力下，我仍能够全神贯注地思考问题	1	2	3	4	5
15	我喜欢在解决问题时起带头作用，而不是让别人主导全局	1	2	3	4	5

16	我不会因失败而气馁	1	2	3	4	5
17	当处理生活中的挑战与困难时，我想我是个坚强的人	1	2	3	4	5
18	必要时，我能做出不合主流的或艰难的决定，而不是随波逐流	1	2	3	4	5
19	我能处理不愉快或痛苦的情绪（如悲伤，害怕和生气）	1	2	3	4	5
20	在处理生活难题时，我不得不按直觉行事	1	2	3	4	5
21	在生活中，我有明确的目标与方向感	1	2	3	4	5
22	我觉得我能掌控自己的生活，而非被外界环境裹挟	1	2	3	4	5
23	我喜欢挑战	1	2	3	4	5
24	不论人生路上遇到什么障碍，我都会努力达到我的目标	1	2	3	4	5
25	我为我取得的成就而感到自豪	1	2	3	4	5

**三、以下是关于数学创造力的描述，看懂即答，请在最符合你实际情况的数字上打“√”。**		完全不符	比较不符	不确定	比较符合	完全符合
1	我喜欢发现和提出数学问题	1	2	3	4	5
2	我喜欢思考，求知欲望强烈	1	2	3	4	5
3	我经常追求有挑战性的数学问题	1	2	3	4	5
4	我喜欢做数学题，并享受解题的快乐	1	2	3	4	5
5	我喜欢做非常规的数学趣味题	1	2	3	4	5
6	碰到很难的数学题，我总是想尽办法把它解出	1	2	3	4	5
7	我能够从数学问题/活动中归纳出教材中要讲的数学概念、定理等	1	2	3	4	5
8	有些数学题，我一看题目就知道怎样解答	1	2	3	4	5
9	我常常会分析、反思、总结自己的解题思路	1	2	3	4	5
10	我常常对同学们都赞同的数学问题有不同的看法	1	2	3	4	5

11	对错误的数学问题，我总能想到办法证明其错误之处	1	2	3	4	5
12	我经常把不熟悉的数学题变成自己熟悉的形式	1	2	3	4	5
13	我能够将已有的解题方法用到新的数学问题中，从而形成自己的想法	1	2	3	4	5
14	我常常从不同角度来理解数学问题	1	2	3	4	5
15	做数学题时，我常常会有好几种解法	1	2	3	4	5
16	对一般数学题，我也经常有独特的解法	1	2	3	4	5
17	我能够全面考虑数学问题，分析可能出现的各种情况	1	2	3	4	5
18	我很熟悉数学开放题，对这类题目有好的解法或思路	1	2	3	4	5
19	我总能注意到数学问题中隐藏的条件，并提出新的问题	1	2	3	4	5
20	对于分类型问题，我总能想出很多种的分类方法	1	2	3	4	5
21	对结论不确定的问题，我总能找到几种不同的结论	1	2	3	4	5
22	我会常常思考老师的教学方法是否适合自己	1	2	3	4	5
23	我会经常反思自己的学习方法，并适时调整	1	2	3	4	5
24	我认为数学在生活中起到很重要的作用	1	2	3	4	5
25	在读课外书时，如果发现里面有很多数字，我总喜欢去找这些数字表示的意思或这些数字的规律	1	2	3	4	5
26	在生活中遇到与数学相关的场景，我总是下意识地用数学方法解决	1	2	3	4	5

**四、以下是关于创造力自我效能感的描述，看懂即答，请在最符合你实际情况的数字上打“√”。**		完全不符	比较不符	不确定	比较符合	完全符合
1	我清楚地知道，我具备有效解决复杂问题的能力	1	2	3	4	5
2	我从不怀疑我的创新能力、创造能力	1	2	3	4	5
3	与同学相比，我有与众不同的想象力和独创性	1	2	3	4	5
4	在很多情况下，我都展现出了我克服困难的能力	1	2	3	4	5
5	我敢保证，我能够解决好需要创新性思维的问题	1	2	3	4	5
6	我非常擅长解决问题时，给出独一无二的原创性答案	1	2	3	4	5
